# Correction: Sato-Akushichi et al. Choroidal Volume Evaluation after Photodynamic Therapy Using New Optical Coherence Tomography Imaging Algorithm. *Pharmaceuticals* 2021, *14*, 1140

**DOI:** 10.3390/ph15030349

**Published:** 2022-03-14

**Authors:** Miki Sato-Akushichi, Shinji Ono, Gerd Klose, Youngseok Song

**Affiliations:** 1 Department of Ophthalmology, Asahikawa Medical University, Asahikawa 078-8510, Japan; o-shinji@asahikawa-med.ac.jp (S.O.); ysong@asahikawa-med.ac.jp (Y.S.); 2 Carl Zeiss Meditec, Inc., Dublin, CA 94568, USA; gerd.klose@zeiss.com

**Error in Figure**

In the original publication [1], there was a mistake in ***Figure 2*** as published. **A boxplot at 3 months of average choroidal thickness in the central area was not printed in *Figure 2*.** The corrected ***Figure 2*** appears below. 

The authors apologize for any inconvenience caused and state that the scientific conclusions are unaffected. This correction was approved by the Academic Editor. The original publication has also been updated.

## Figures and Tables

**Figure 2:**
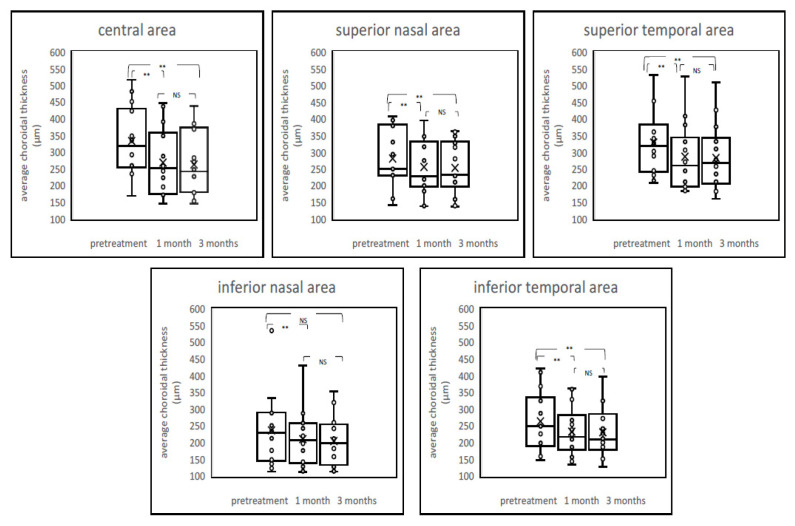
Boxplot graphs of average choroidal thickness before and after treatment. The post-treatment average choroidal thickness showed a significant reduction from baseline at all time points after the PDT, including both the irradiated central area and nonirradiated peripheral areas. NS = not significant, ** *p* < 0.01.
