# Supplementary material for: Choroidal Volume Evaluation after Photodynamic Therapy Using New Optical Coherence Tomography Imaging Algorithm
Source: Pharmaceuticals (Basel). 2021 Nov 10;14(11):1140. doi: 10.3390/ph14111140 (PMC8621234; doi:10.3390/ph14111140)
Supplement: Supplementary file 1 [file pharmaceuticals-14-01140-s001.zip › pharmaceuticals-1439820-SI.pdf]

Supplementary Table S1. Choroidal volume between patients before and after the PDT and control

		normal control	CSC		
			**pretreatment**	**one month**	**three months**
**choroidal volume**	**central area**	1.65 (0.63)	2.36 (0.70) *p*=0.039	1.90 (0.69) *p*<0.001	1.86 (0.66) *p*<0.001
	**superior nasal area**	0.92 (0.26)	1.25 (0.38) *p*=0.096	1.14 (0.35) *p*=0.002	1.13 (0.34) *p*=0.002
	**superior temporal area**	1.13 (0.31)	1.47 (0.41) *p*=0.096	1.28 (0.43) *p*<0.001	1.26 (0.43) *p*<0.001
	**inferior nasal area**	0.82 (0.26)	1.07 (0.49) *p*=0.224	0.95 (0.38) *p*=0.013	0.93 (0.35) *p*=0.062
	**inferior temporal area**	1.03 (0.36)	1.17 (0.38) *p*=0.305	1.04 (0.32) *p*<0.001	1.03 (0.33) *p*=0.001

Data are presented as mean (standard deviation) and round off to three decimal places.

Mann-Whitney U test for the comparison between central serous chorioretinopathy (CSC) pretreatment and normal control and Bonferroni test for the comparison between CSC pretreatment and one month, pretreatment and three months after the treatment. *p* values written in CSC pretreatment section were compared to values in normal control and written in CSC one month and three months section were compared to values in CSC before treatment.

Supplementary Table S2. Average choroidal thickness, maximum and minimum choroidal thickness between patients before and after the PDT and control

		normal control	CSC		
			pretreatment	one month	Three months
**Average choroidal thickness**	**central area**	233.52 (89.53)	333.96 (98.52)<br>*p*=0.039	269.09 (97.40)<br>*p*<0.001	263.67 (92.54)<br>*p*<0.001
	**superior nasal area**	206.97 (57.48)	281.73 (84.43)<br>*p*=0.096	255.71 (78.93)<br>*p*=0.002	253.16 (77.09)<br>*p*=0.002
	**superior temporal area**	253.69 (69.79)	328.98 (93.14)<br>*p*=0.096	286.59 (96.58)<br>*p*<0.001	283.34 (96.05)<br>*p*<0.001
	**inferior nasal area**	183.12 (58.83)	236.38 (105.25)<br>*p*=0.224	210.12 (82.87)<br>*p*=0.001	204.97 (72.01)<br>*p*=0.069
	**inferior temporal area**	231.06 (80.59)	263.53 (86.23)<br>*p*=0.305	232.56 (71.96)<br>*p*<0.001	230.54 (73.37)<br>*p*=0.001
**maximum choroidal thickness**	**central area**	302.39 (119.61)	407.30 (103.43)<br>*p*=0.072	341.62 (113.70)<br>*p*=0.003	316.99 (122.86)<br>*p*=0.005
	**superior nasal area**	271.99 (77.79)	355.67 (97.32)<br>*p*=0.053	316.45 (100.21)<br>*p*=0.001	319.46 (104.79)<br>*p*=0.021
	**superior temporal area**	298.96 (81.65)	373.92 (97.47)<br>*p*=0.096	328.49 (103.40)<br>*p*<0.001	322.43 (98.90)<br>*p*<0.001
	**inferior nasal area**	235.72 (80.20)	296.99 (120.87)<br>*p*=0.201	263.05 (102.10)<br>*p*=0.001	252.86 (86.52)<br>*p*=0.024
	**inferior temporal area**	286.53 (94.68)	330.95 (92.46)<br>*p*=0.224	289.18 (79.01)<br>*p*<0.001	287.37 (75.10)<br>*p*<0.001
**minimum choroidal thickness**	**central area**	160.21 (51.58)	246.41 (93.40)<br>*p*=0.046	195.54 (87.65)<br>*p*<0.001	192.21 (83.83)<br>*p*<0.001
	**superior nasal area**	154.28 (45.44)	213.44 (75.08)<br>*p*=0.053	200.80 (64.89)<br>*p*=0.103	197.53 (62.81)<br>*p*=0.026
	**superior temporal area**	200.75 (47.71)	258.11 (97.98)<br>*p*=0.224	225.52 (89.64)<br>*p*=0.003	228.50 (89.41)<br>*p*=0.014
	**inferior nasal area**	142.20 (42.29)	177.76 (86.36)<br>*p*=0.369	157.51 (55.23)<br>*p*=0.16	153.72 (46.14)<br>*p*=0.28
	**inferior temporal area**	176.36 (61.69)	200.38 (72.93)<br>*p*=0.516	181.09 (62.20)<br>*p*=0.011	177.35 (62.11)<br>*p*=0.011

Data are presented as mean (standard deviation) and round off to three decimal places. Mann-Whitney U test for the comparison between central serous chorioretinopathy (CSC) pretreatment and normal control and Bonferroni test for the comparison between CSC pretreatment and one month, pretreatment and three months after the

treatment. *p* values written in CSC pretreatment section were compared to values in normal control and written in CSC one month and three months section were compared to values in CSC before treatment.

Supplementary Tables S3

Supplementary Table S3.1: The demographics between three different treatment groups

	Reduced dose (n=5)	Reduced fluence (n=7)	Reduced time (n=2)	*p* value
**Age**	59.8	64.6	41.0	0.086
**logMAR (pretreatment)**	0.162	0.210	-0.079	0.234
**logMAR (one month)**	0.184	0.248	0.071	0.417
**logMAR (three months)**	0.152	0.237	-0.079	0.222
**Mean refractive error**	0.15	-0.54	0.25	0.980
**spot size**	4620	4529	4250	0.978

Data are presented as mean and the Kruskal-Wallis test for the comparison between three groups.

Supplementary Table S3.2: Choroidal Volume between three different treatment groups before and after the PDT

		Reduced dose	Reduced fluence	Reduced time	*p* value
**Central area**	**pretreatment**	2.229	2.188	3.305	0.188
	**one month**	1.771	1.694	2.969	0.128
	**three months**	1.842	1.596	2.855	0.153
**Superior nasal area**	**pretreatment**	1.286	1.079	1.789	0.093
	**one month**	1.172	0.962	1.680	0.052
	**three months**	1.197	0.962	1.538	0.133
**Superior temporal area**	**pretreatment**	1.556	1.199	2.193	0.032*
	**one month**	1.318	1.024	2.081	0.025*
	**three months**	1.364	0.986	1.969	0.025*
**Inferior nasal area**	**pretreatment**	1.110	1.045	1.057	0.632
	**one month**	0.985	0.927	0.927	0.744
	**three months**	1.025	0.869	0.884	0.661
**Inferior temporal area**	**pretreatment**	1.184	1.133	1.288	0.618
	**one month**	1.046	1.010	1.097	0.694
	**three months**	1.077	0.985	1.045	0.756

Data are presented as mean and round off to four decimal places. The Steel-Dwass test was used for multiple comparisons in each area between different treatment conditions. No statistically significant difference was found among superior temporal area at all time even though there were statistically significant differences in Kruskal-Wallis test.

Supplementary Table S3.3: Average choroidal thickness between three different treatment groups before and after the PDT

		Reduced dose	Reduced fluence	Reduced time	*p* value
**Central area**	**pretreatment**	315.12	309.34	467.20	0.188
	**one month**	250.33	239.45	419.73	0.128
	**three months**	260.45	225.98	403.63	0.153
**Superior nasal area**	**pretreatment**	288.84	242.33	401.85	0.093
	**one month**	262.71	216.00	377.21	0.052
	**three months**	268.24	216.04	345.36	0.106
**Superior temporal area**	**pretreatment**	349.58	267.55	492.48	0.032*
	**one month**	295.97	228.27	467.28	0.025*
	**three months**	306.44	221.44	442.26	0.025*
**Inferior nasal area**	**pretreatment**	238.19	234.78	237.45	0.632
	**one month**	213.48	208.26	208.25	0.744
	**three months**	221.32	195.11	198.58	0.744
**Inferior temporal area**	**pretreatment**	265.98	254.43	289.26	0.618
	**one month**	234.92	226.92	246.40	0.694
	**three months**	242.00	221.17	234.66	0.756

Data are presented as mean and round off to three decimal places. The Steel-Dwass test was used for multiple comparisons in each area between different treatment conditions. No statistically significant difference was found among superior temporal area at all time even though there were statistically significant differences in Kruskal-Wallis test, as well as choroidal volume.

Supplementary Table S3.4: Maximum choroidal thickness between three different treatment groups before and after the PDT

		Reduced dose	Reduced fluence	Reduced time	*p* value
**Central area**	**pretreatment**	377.18	392.49	534.46	0.072
	**one month**	319.23	308.96	511.91	0.049*
	**three months**	331.54	258.03	487.01	0.025*
**Superior nasal area**	**pretreatment**	371.59	302.99	500.24	0.025*
	**one month**	323.50	262.61	487.21	0.008*
	**three months**	331.36	275.44	443.75	0.059
**Superior temporal area**	**pretreatment**	386.43	317.31	540.78	0.012*
	**one month**	326.79	276.01	516.41	0.012*
	**three months**	338.30	265.97	480.34	0.012*
**Inferior nasal area**	**pretreatment**	297.76	292.02	312.50	0.402
	**one month**	261.26	260.70	275.77	0.619
	**three months**	273.00	235.95	261.67	0.513
**Inferior temporal area**	**pretreatment**	337.68	321.96	345.61	0.588
	**one month**	291.11	285.38	297.67	0.296
	**three months**	305.31	275.10	285.48	0.393

Data are presented as mean and rounded off to three decimal places. The Steel-Dwass test was used for multiple comparisons. In the central area, there was no statistically significant difference between the one month and three months after treatment (p value > 0.05). However, in the superior nasal area, there were statistically significant differences between the half fluence group and the half time group before and one month after treatment (p=0.038, respectively). There was also a statistically significant difference between the half fluence group and the half time group at all time in the superior temporal area (p=0.038, respectively).

Supplementary Table S3.5: Minimum choroidal thickness between three different treatment groups before and after the PDT

		Reduced dose	Reduced fluence	Reduced time	*p* value
**Central area**	**pretreatment**	238.06	215.59	375.15	0.086
	**one month**	187.16	161.83	334.49	0.030*
	**three months**	190.15	158.28	316.12	0.028*
**Superior nasal area**	**pretreatment**	228.68	183.58	279.89	0.122
	**one month**	213.34	174.33	262.10	0.122
	**three months**	217.70	171.58	237.95	0.102
**Superior temporal area**	**pretreatment**	269.38	203.08	422.53	0.015*
	**one month**	228.82	175.69	391.68	0.011*
	**three months**	236.64	180.24	377.10	0.022*
**Inferior nasal area**	**pretreatment**	167.39	186.48	173.15	0.846
	**one month**	159.07	157.38	154.05	0.588
	**three months**	160.85	150.74	146.33	0.543
**Inferior temporal area**	**pretreatment**	193.95	197.57	226.28	0.524
	**one month**	176.36	178.69	201.34	0.524
	**three months**	180.60	174.35	179.75	0.866

Data are presented as mean and rounded off to three decimal places. The Steel-Dwass test was used for multiple comparisons in each area between different treatment conditions. In the central area, there was no statistically significant difference between the one month and three months after treatment (p value > 0.05). However, there was a statistically significant difference between the half fluence group and the half time group at all time in the superior temporal area (p=0.038, respectively).
